# Migraine, interferon β1a and siponimod immunomodulator therapies

**DOI:** 10.1186/s12871-022-01639-z

**Published:** 2022-04-05

**Authors:** Ashraf A. Dahaba, Helmar Bornemann-Cimenti

**Affiliations:** 1 Department of Anaesthesiology and Intensive Care Medicine, Suez Canal University, 41522 Ismailia, Egypt; 2 Department of Anesthesiology and Intensive Care Medicine, Medical University of Graz, Auenbruggerplatz 5/5 Graz, Styria 8036 Graz, Austria

**Keywords:** Migraine, Headache, Premonitory symptoms, Multiple sclerosis, Interferon β1a, Siponimod, Immunomodulator

## Abstract

**Background:**

Autoimmunity seems to play a great role in the pathogenesis of migraine headache pain. There is far more evidence that interferon can exacerbate migraines. We report a case where remission of severe comorbid migraine attacks happened with the start of interferon β1a (Merck, Netherlands) immunomodulation therapy. Therapy for multiple sclerosis was decided according to the severity of the debilitating comorbid migraine headache pain rather than the evolution of multiple sclerosis the far more serious disease.

**Case presentation:**

A 63-years old patient suffered for 30-years from migraine headache of severe disability assessment scale (MIDAS) Grade-IV = 27. He also suffered for 25-years from optic-sensory relapsing remitting multiple sclerosis (RRMS). Subcutaneous interferon β1a 44-µg immunomodulation therapy for 4-years resulted in multiple sclerosis complete remission. The start of interferon β1a therapy for multiple sclerosis seemed to help resolving the comorbid migraine attacks. The visual aura premonitory symptom preceding migraine headache would end up with a feeling of post visual aura clearer field of vision and a feeling of wellbeing. As the patient developed secondary progressive multiple sclerosis (SPMS), oral siponimod 2 mg (Novartis, Ireland), currently the only available therapy for SPMS, replaced his interferon therapy. This was associated with a relapse of migraine severe attacks. Reverting back to interferon therapy was again associated with migraine headache remission.

**Conclusions:**

Interferon β1a might be an efficic therapy for “autoimmune migraine”. With numerous immunomodulators currently available for other systemic autoimmune diseases associated with comorbid migraine; examining the effect of these immunomodulatory therapies on comorbid migraine headache could be beneficial in finding a specific immunomodulator therapy for “autoimmune migraine”.

## Background

In an ongoing debate regarding the role of autoimmunity in migraine headache pathophysiology; immunological dysfunction seems a plausible pathogenesis of migraine headache pain. Among the complex relationship between interferon and migraine; there seems to be far more evidence that interferon can exacerbate migraines 1. In our patient the start of interferon β1a (Merck, Netherlands) immunomodulation therapy for multiple sclerosis, seemed to help resolving the comorbid migraine attacks. Therapy for multiple sclerosis, the far more serious disease, was decided according to the severity of the debilitating comorbid migraine headache rather than the progression of multiple sclerosis.

## Case presentation

A 63-yers old patient, who gave a written informed consent that we can use his data, suffered for 30 years from migraine headache of severe migraine disability assessment scale (MIDAS) 2 Grade IV = 27 that was preceded by 30 min visual aura premonitory symptom. In addition to migraine maternal family history of similar intensity and frequency extending back for 2 generations, patient also suffered from 2 systemic autoimmune diseases namely Hashimoto’s disease and for 25 years from optic-sensory relapsing remitting multiple sclerosis (RRMS) of expanded disability status scale (EDSS) = 1.5. Subcutaneous interferon β1a 44 µg immunomodulation multiple sclerosis therapy for 4 years resulted in complete RRMS remission. The start of interferon β1a immunomodulation therapy seemed to help resolving the severe comorbid migraine attacks; as the 30 min visual aura premonitory symptom preceding the migraine headache would now often end with a feeling of post visual aura clearer field of vision and a feeling of wellbeing.

Without new RRMS relapses, patient’s condition gradually deteriorated to 5.5 EDSS secondary progressive multiple sclerosis (SPMS) rendering him immobile. A mandatory pharmacogenetic analysis revealed no mutations in the CYP3A4 or CYP29C genes responsible for siponimod (Novartis, Ireland) excretion; patient switched from s.c. interferon β1a to oral siponimod 2 mg, currently the only licensed therapy for SPMS. Migraine severe attacks recurred similar in intensity and frequency to before the interferon therapy.

With severe migraine headache relapse, it was decided that interferon β1a would be concomitantly administered with oral siponimod therapy for 1 week. Patient experienced no migraine attaches during that week. After written informed consent; the patient was made fully aware that the simultaneous discontinuation of the 2 immunomodulators interferon β1a and siponimod for a 2-week “migraine test period” could expose him to multiple sclerosis relapses or formation of new demyelination cerebral lesions. The 2-week “migraine test period” was associated with recurrence of migraine attacks similar in intensity and frequency to before the interferon therapy. Reverting back to interferon β1a once more was associated with migraine remission that lasted for 2 years to-date. The patient is currently on long-term follow up for possible future migraine attacks.

## Discussion

This is an interesting case describing the possibility of an interferon β1a immunomodulator helping to resolve migraine attacks in a known multiple sclerosis patient. This speculation adds to the ongoing research on the potential of neurogenic inflammation for migraine pathogenesis. Immunomodulator therapies might have far reaching influence on migraine headache pain. There is more evidence that interferon can exacerbate migraine headache. 1 We report a case where relief of severe comorbid migraine attacks occurred with the start of interferon β1a immunomodulation therapy. When interferon “immunomodulation cover” dissipated during the siponimod replacement therapy or during the migraine test period, in both occasions this was associated with severe migraine headache relapse. With resumption of interferon therapy the patient experienced no migraine attaches; indicating that interferon β1a but not siponimod immunomodulation could be an efficic therapy for “autoimmune migraine”.

Scientists have long investigated the pathophysiology of migraine headache pain and debated various mechanisms of the pathogenesis that can generate the state of “sterile inflammation” in the intracranial meninges, 3 sensitizing the meningeal afferents densely innervating the dural vasculature, result in nociceptors activation, vasodilation, plasma extravasation, edema and mast cell degranulation. 4 With interferon β1a therapy the 30 min visual aura premonitory symptom that preceded the migraine headache often did not end with migraine headache pain rather with a feeling of post visual aura clearer field of vision and a feeling of wellbeing.

There has been a debate over the role of autoimmunity in migraine pathophysiology when different immunomodulators could either ameliorate or exacerbate migraine headaches. 1 We advocate that immunological dysfunction and/ or autoimmunity is a plausible pathophysiology of migraine 3 as our patient has two comorbid systemic autoimmune diseases. Large propensity cohort population–based studies suggest an association between the two pathologies. Systemic autoimmune diseases such as lupus erythematosus, antiphospholipid syndrome, Sjogren’s syndrome and systemic sclerosis are 2-3 fold more frequent in patients suffering from migraine. 5 A recent epidemiological study in male patients with multiple sclerosis demonstrated the high rate of comorbid migraine in 27% of patients. 6 A non-systemic literature review 3 tried to substantiate a novel hypothesis of autoimmune migraine pain; as migraine patients had reduced regulatory T cells in peripheral blood, incriminating an exaggerated immune response; a strong evidence of immunological dysfunction and/or autoimmunity. 3 Whereas administration of immunotherapy significantly decreased the prevalence, frequency, and disability of migraine headache in younger subjects. 7.

In our patient, the temporal association between the onset of migraine attacks and the onset of multiple sclerosis is close, with migraine headache starting just few years before the start of multiple sclerosis. That makes it difficult to delineate whether the migraine is de novo primary headache or whether this is secondary to multiple sclerosis. The migraine headache could have been an early presenting feature of multiple sclerosis. Here a randomized clinical trial and longitudinal studies are needed to analyse the cause-effect relationships between the headaches (which could be due to migraine) and multiple sclerosis.

We cannot totally exclude the role of the immunomodulator siponimod in provoking patient’s migraine attacks when interferon β1a was discontinued. A previous *Lancet Neurology* 8 study reported migraine as a side effect in patients with RRMS, whereas a recent *Lancet* 9 publication mentioned headache and not specifically migraine as one of siponimod side effects.

## Clinical Implications

1. Subcutaneous Interferon β1a 44 μg multiple sclerosis therapy was associated with relief of severe migraine attacks.

2. The visual aura premonitory symptom that used to precede migraine headache pain would frequently end up with a feeling of post visual aura clearer field of vision and a feeling of wellbeing instead of migraine headache pain.

3. Oral siponimod 2 mg, secondary progressive multiple sclerosis immunomodulator replacement therapy was associated with relapse of migraine severe attacks similar to those beforeinterferon therapy.

4. Reverting back to interferon therapy was associated with remission of the migraine headache attacks.

5. Interferon β1a but not siponimod immunomodulation might be an efficic therapy for “autoimmune migraine”.

## Conclusions

Interferon β1a but not siponimod immunomodulation could be an efficic therapy for “autoimmune migraine”. With numerous immunomodulatory therapies currently available for multiple sclerosis and other systemic autoimmune diseases in patients suffering from comorbid migraine; examining the effect of these long-term immunomodulatory therapies on comorbid migraine headache could be beneficial in finding a specific immunomodulator therapy for “autoimmune migraine”.

## Data Availability

Not applicable, as our manuscript does not contain any additional data other than reported data. Both authors declare that all relevant material are readily available.

## Abbreviations

MIDAS: Migraine disability assessment scale
RRMS: Relapsing remitting multiple sclerosis
EDSS: Expanded disability status scale
SPMS: Secondary progressive multiple sclerosis
